# Role of exosomes and exosomal microRNAs in cancer

**DOI:** 10.2144/fsoa-2019-0116

**Published:** 2020-02-26

**Authors:** Nihat Dilsiz

**Affiliations:** 1 Department of Molecular Biology & Genetics, Faculty of Engineering & Natural Sciences, Istanbul Medeniyet University, Istanbul, Turkey

**Keywords:** biomarkers, body fluids, cancer, exosomes, extracellular vesicles, miRNA

## Abstract

A growing body of evidence indicates that exosomes play a critical role in the cell–cell communication process. Exosomes are biological nanoparticles with an average diameter of 30–100 nm in size and are produced by almost all cell types in the human body; however, cancer cells contain higher concentrations of exosomes than healthy cells. They are released into all body fluids and contain double-stranded DNA (originated from nucleus and mitochondria), a variety of RNA species, and specific protein biomarkers that can be utilized as cancer biomarkers and therapeutic targets, and lipids. Therefore, the specific exosomes secreted by tumor cells could be used to predict the existence of the presence of a tumor in cancer patients. This review summarizes the role of exosomes in cancer development and their potential utility in the clinic.

## Lay abstract

Intercellular communication between tumor cells and their neighboring cells and distant organs is key to cancer’s survival, progression, drug resistance and metastasis. Circulating exosomes carry a specific composition of proteins, lipids, RNA and DNA and can work as cargo to transfer this information from donor cells to target cells, leading to reprogramming of the recipient cells. Exosomes are small particles with big roles in cancer. The present review summarizes the significant aspects of exosomes in cancer development and how they might provide novel strategies for use in the clinic.

Cancer is the second highest cause of death and a major health problem worldwide, with approximately 18 million new cases and 9.6 million deaths annually; this is 16.5% of human deaths that occurred in 2017 according to WHO [1]. More than half of the world’s population will be afflicted by invasive cancer at some point during their lifetime [2]. Early-stage cancer diagnosis is key for positive prognosis, which can extend lifespan and reduce the number of disease-related death [3,4]. Cancer can have around 20 years of incubation period before it is detectable by ultrasound, x-ray-based computer tomography, endoscopy or other detection methods including tissue biopsy. However, the new and highly sensitive detection method, liquid biopsy-based exosome analysis, provides a promising platform for early diagnosis, therapeutic and prognostic process about a disease rather than the conventional tissue biopsy. Liquid biopsy, a new star of cancer detection, is growing in popularity because of their minimal invasiveness, ease of use, painless, lower sample volume, lower cost, more accuracy and high throughput for personalized cancer therapy [5,6,7]. It is important to know that most cancers can be more effectively treated if they are discovered in early stages.

Cell communication and transformation are essential in tumorigenesis: single tumor cells must interact with each other and with nontumor host cells to enhance tumor cell growth, survive, progress, angiogenesis and metastasis [8]. It is becoming increasingly clear that tumor cell-derived exosomes play a key role in this communication process through the transfer of various biomolecules including proteins, lipids, DNA and RNA, which can be transferred in active form from the donor to the recipient cells [9]. They are extremely stable and resistant against degradation enzymes such as RNases and can keep their contents intact for a longer time than other materials such as cells. 

Almost all cancer cells harbor aberrant expressions of microRNAs (miRNAs), that a key component of the small noncoding RNA family. These cancer-specific upregulated or downregulated short noncoding miRNAs are extremely important for the cancer development by altering oncogenes, tumor suppressor genes and therefore cancer-related signalling pathways. Onco-miRNAs are aberrantly expressed in cancer cells and target the degradation of key tumor suppressor messenger RNA to promote tumor formation or growth. On the other hand, the elevated expressions of tumor suppressive miRNAs inhibit tumor growth by inactivating expressions of oncogenes and hence are in general downregulated in cancer cell growth [10].

## Exosomes & microvesicles

Almost all our body’s cells release a various types of nanometer-sized membrane-derived lipid bilayer vesicles into the extracellular environment, which are collectively termed as extracellular vesicles (EVs) [1,12]. EVs have been detected in a various biological fluid, including saliva, amniotic fluid, breast milk, semen, nasal secretion, cerebrospinal fluid, lymph, tear, aqueous humor, urine and blood plasma or serum.

These membrane-derived EVs can be classified into three different classes depending on their cellular origin, sizes and dimensions, mode of release, their contents, and functions. These are named as exosomes (∼30–100 nm), microvesicles (∼100–1000 nm) and apoptotic bodies (∼500–3000 nm). Exosomes were first discovered by Pan and Johnstone in 1983 [13].

**Figure 1.**
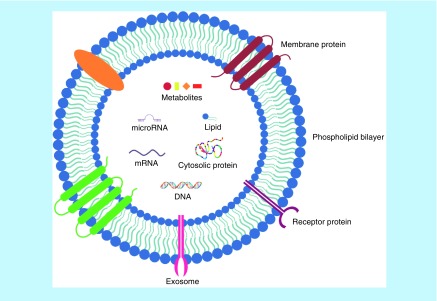
The structure and content of exosome. Exosomes contain various types of proteins, nucleic acids, lipids and metabolites.

Differently from the other cellular vesicles such as apoptotic bodies (highly heterogeneous in size and composition) that formed at the final stages of apoptosis from the plasma membrane, exosomes are of endocytic origin and microvesicles (ectosomes, shedding vesicles) are formed a direct outward budding of the cell’s plasma membrane. Structurally, exosomes are the smallest EVs, display a cup-like shape when examined by transmission electron microscopy and are more homogeneous in shape than the other EVs (Figure 1). Exosomes are formed as intraluminal vesicles by a process that involves the endocytic pathway and are secreted upon fusion of late endosomes or multivesicular bodies (MVBs) with the plasma membrane and are released into the extracellular space (exocytosis) [14,15,16,17,18,19,20,21].

**Figure 2.**
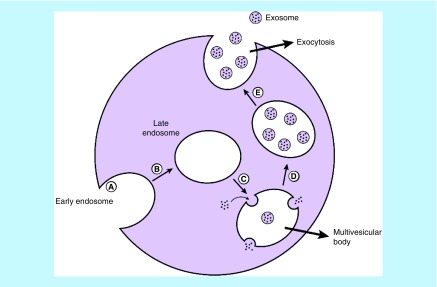
The formation and releasing of exosome. **(A)** Exosome is derived from early endosome formed from plasma membrane. **(B)** Early endosome becomes late endosomes. **(C)** Then forms early multivesicular bodies. **(D)** Late multivesicular bodies. **(E)** Late multivesicular bodies can either get degraded by lysosomes or fuse with the membrane to release exosomes.

The formation of exosomes can be categorized into three different stages: formation of early endocytic vesicles from plasma membrane (early endosome), inward-budding of the endosomal vesicle membrane resulting in MVBs that consist of ILVs and fusion of these MVBs with either lysosome in which they are degraded, or the plasma membrane, which releases the vesicular contents, known as exosomes (late endosome) (Figure 2) [22]. During the exosome formation, cytoplasmic biomolecules including nucleic acids and proteins are trapped inside lumen. The contents of exosomes are sorted and loaded through either endosomal-sorting complexes required for transport system (ESCRT)-dependent (in cooperation with apoptosis-linked gene 2 interacting protein X (ALIX) and tumor-susceptibility gene 101 protein) or an ESCRT-independent (with tetraspanins proteins and lipids such as sphingosine-1-phosphate and ceramide dependent) mechanisms [23,24].

Both healthy and cancerous cells may release membrane-bound exosomes into the extracellular space and body fluids. However, cancer cells can produce about tenfold more exosomes when compared with normal healthy cells. The entry of exosome into recipient cells usually is made up the processes called endocytosis that can be measured by using methods such as confocal microscopy or flow cytometry. Endocytosis is an umbrella term for a range of molecular internalization pathways in the uptake of exosomes [25].

**Figure 3.**
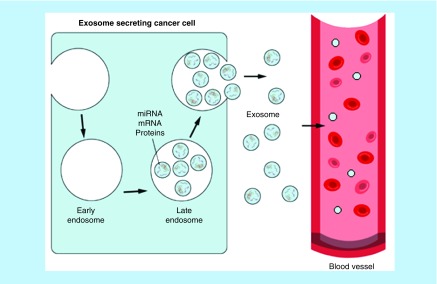
Exosomes biogenesis and sorting into the blood. Exosome can be either fused with lysosomes for degradation or with plasma membrane thereby releasing exosomes to the extracellular space. Cell-released exosomes then can be taken up by neighboring recipient cells or travel through biological fluids such as blood, urine or saliva.

**Figure 4.**
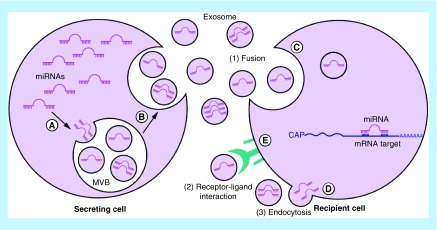
Transfer of exosomal miRNA from donor cell to recipient cells. **(A)** miRNAs are sorted to exosome during MVB formation. **(B)** Exosomes are released into the extracellular space. **(C)** Exosomal miRNAs can be delivered to recipient cells by endocytosis. **(D)** Fusion of the exosomes with the plasma membrane using soluble N-ethylmaleimide sensitive fusion into cells primarily uses receptor-mediated endocytosis. **(E)** Exosomes may also bind to a receptor and activate specific signaling pathways. MVB: Multivesicular body.

They are released from tumor cells (tumor-derived exosomes) into their surrounding extracellular space, and growing evidence indicates that these vesicles have multi functions including initiation of tumor progression, immune suppression, neovascularization, metastasis and drug resistance [26]. Exosomes are valuable sources for biomarker researches, as their contents are a wealth of information on the state of their cell of origin and function in biological processes and they are released in all biological fluids, including blood, tear, urine and saliva (Figure 3). Exosomes are also recognized as important mediators in cell to cell communication by transferring their contents (Figure 4). Indeed, many studies have now found evidence that exosomal contents, including double-stranded DNA, a variety of RNA species and specific protein biomarkers that are important as cancer predictive biomarkers for early cancer diagnosis and determination of prognosis. A total of 4.960 articles related to exosomes were identified in the study period from 1997 to 2017 by using Scopus and Web of Science [21].

## Biological function of exosomes in cancer

Exosomes play a fundamental biological role in intercellular communication, induce physiological changes in recipient cells by transferring their cargo and have been implicated in many diseases such as cancer, cardiovascular diseases, autoimmune syndromes, neurodegenerative disorders and many others [2,27]. Tumor cell-released exosomes induce alterations in their recipient cells, thereby playing a crucial role in promotion of primary tumor development, stimulation of angiogenesis, activation of stromal fibroblasts, sculpting the cancer extracellular matrix adhesion, promotion of a premetastatic niche formation, suppression of the host immune response, resisting cell death and developing drug-resistance [17,28]. Tumor cell-released exosomes are also participating in the development of drug resistance to anticancer therapies and stimulate secretion of antiapoptotic proteins in tumor cells.

Exosomes contain various proteins such as ESCRT (ESCRT 0, I, II and III; which are required for transport), tetraspanins (transmembrane proteins induce vesicle formation), Rab GTPases (Rab7/Rab9/Rab11/Rab27/Rab35; which are essential for exosome release, tumor growth and metastasis), heat-shock proteins (HSP20/HSP60/HSP70/HSP90) and transforming growth factor β. Selecting, binding and uptaking of exosomes to the surface of recipient cells is mediated by different proteins such as tetraspanin family proteins (TSPAN: CD9, CD37, CD49, CD53, CD63, CD81 and CD82), immunoglobulins, proteoglycans, lectins and intercellular adhesion molecules (e.g., integrins with alpha subunits [ITGA], and with beta subunits [ITGB]) on the surface of both the exosome and the target cell. These attachments can then lead to direct delivery of exosomal cargo molecules into the recipient cell at new locations, and conceivably changing their biology (Table 1) [10,17,29,30,31].

**Table 1.** Biological properties of some extracellular vesicles.

Extracellular vesicles	Exosomes	Microvesicles (microparticles)	Apoptotic bodies
Size	30–100 nm	100–1000 nm	500–3000 nm
Shape	Cup shaped	Irregular	Heterogeneous
Sedimentation	100,000 × g or higher	1200–100,000 × g	1200–100,000 × g
Biomarkers	ALIX, TSG101, ESCRT-0, -I, -II and -III, tetraspanins-TSPAN/CD9/CD24/CD49/CD63/CD53/CD81/CD82, heat shock proteins- HSP20/HSP60/HSP70/HSP90, flotillins, GTPases- Rab11/Rab27/Rab31/Rab35.	Integrins-CD40/CD51/CD61, ligand, flotillin-2, metalloproteinase, selectins	Histones, annexin V positivity adhesion molecules, chemokines
Mode of release	Exocytosis, multivesicular endosomes	Exocytosis	Condensed apoptotic fragments

ESCRT: Endosomal-sorting complexes required for transport system; TSG101: Tumor-susceptibility gene 101 protein.

**Figure 5.**
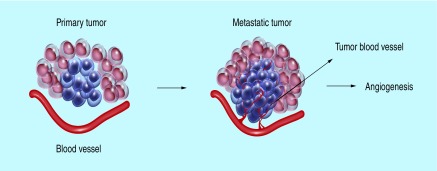
Primary tumor and angiogenesis. **(A)** Cell proliferate to form primary tumor formation (without blood vessels). **(B)** Tumor mass increase and produce angiogenic factors that stimulate new blood vessel formation from the main blood vessel toward the tumor cells.

Exosomes can act as carriers to transport oncogenic proteins and nucleic acids from donor tumor cell to normal recipient target cells at distance from the originating cell. These horizontal (lateral) molecular transfers of exosomal factors can modulate cell signalling pathways in transformed and even untransformed cells. The formation of new blood vessel (angiogenesis) is an important part of preparing a site for future colonization by cancer cells (Figure 5). To achieve this, cancer cells secrete exosomes that act in diverse ways to induce neoangiogenesis at their premetastatic niche, and promoting cancerous cell migration [10]. It has been demonstrated that tumor cell-released exosomal miRNAs such as miR-9, miR-23a, miR-92a, miR-103, miR-105, miR-126, miR-132 miR-135b, miR-210, miR-221 and cytokines (e.g., interleukins: IL-6 and IL-8, TNF-α, transforming growth factor β, FGF2, and VEGF) are proangiogenic factors to promote neovascularization and metastasis [32,33,34,35,36,37,38]. For example, exosomal miR-9 secreted by tumor cells, activates the Janus kinase/signal transducers and activators of transcription JAK/SAT pathway by reducing cytocine signaling 5 (SOCS5) levels to promote tumor angiogenesis [39]. Additional work showed that miR-105 induces vascular leakiness and promoting metastasis [33].

Metastasis is the main cause of mortality in cancer patients, accounting for more than 90% of all cancer-related deaths [2,40]. Metastasis is an enormously complex process by which cancer cells originating from a malignant primary tumor spread and colonize in distant organs within the body, establishing secondary tumors in different tissues. Recent studies have shown that tumor cell-derived exosomes play a prominent role in the pathology of tumor metastases by using tumor-signaling pathway such as caveolin-1, HIF-1a, miR-21, miR-105, miR-210, *β*-catenin and oncogenic kinases (e.g., mutated EGFR, RAS and MAP kinases) [2,41,42,43,44].

## Exosome isolation techniques

For an effective use of exosomes as source of biomarker discovery in liquid biopsies, highly pure exosome samples are required [26]. Therefore, the choice of the suitable separation and isolation method is important. Unfortunately, due to their small size with diameters between 30 and 100 nm and low density, exosomes are extremely difficult to define, isolate and purify from other component in the blood plasma and requires major time and effort. There are two crucial points that must be controlled to achieve a high quality in exosome preparations: the appropriate collection and storage of the bodily fluid samples (such as source of fluids, preparation conditions and storage temperature) and the purity and yield of the isolated exosomes.

In blood, plasma is preferred for exosomal microRNAs (exo-miRNAs) analysis, as the preparation of plasma is less complex and slightly easier than that for serum, because serum contains high numbers of vesicles released by platelets in response to coagulation [45,46]. A recent exo-miRNAs analysis of 312 human plasma and serum samples collected from 13 healthy volunteers indicated that 78% of total RNAs in plasma and 53% of total RNAs in serum was exo-miRNAs [47]. Blood plasma is a yellow liquid consists of water with many substances dissolved in it such as: mineral salts and ions, low/high molecular weight components, gases and metabolites, and acts as the extracellular matrix of blood cells. It represents approximately 55% of the body’s total blood volume.

For exosome isolation, collected blood (0.5–1.0 ml) in potassium EDTA-coated tubes should be processed within 30 min after collection. Samples are cool centrifuged at 1500 × *g* for about 10 min to remove dead cells and then at 10,000 × *g* for about 10 min to remove the cellular debris and nonexosomal vesicles. Separated plasma sample aliquots should be used immediately, or stored at around -80°C until use.

**Figure 6.**
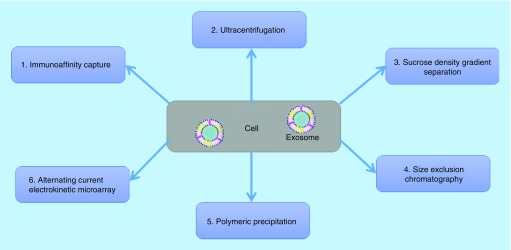
Overview of the different exosome isolation and purification techniques.

In recent years, various conventional protocols have been developed and applied to isolate and purify exosome from bodily fluids and cell culture media: ultracentrifugation-based technique at 100,000 × *g* [48], nano-membrane concentrator-based approach [49], immunoaffinity-based capture using monoclonal antibody-coupled nanobeads [50,51], sucrose density gradient separation using sucrose or Percoll [52], alternating current electrokinetic microarray chip technology (ACE) [3,53], nanowire-anchored microfluidic platforms [54,55,56] and utilizing a commercially available synthetic polymer-based precipitation reagents (Figure 6) [57,58]. Each of these methods has their own advantages and disadvantages for exosome isolation and purification from various biological samples. The ultracentrifugation-based technique is the classical and most commonly used (over 80%) isolation method. Overall, the preanalytical steps such as sample collection, storage, exosome concentration and processing time are important for the efficient and reliable method for the analysis of exosomes.

Although advances have been used to isolate and analyze exosome miRNA, there remains a need for a rapid, sensitive and cost-effective gold standard method that generates an effective, pure isolation, detection, high yield extraction and accurate quantification of exo-miRNA from body fluids for research. This is because of the extremely low concentration of exo-miRNAs in body fluids (<0.01%) [59].

## Physical characterization & molecular analysis techniques for exosome

Due to their small size (30–100 nm), accurate quantification and characterization of exosomes is technically challenging. Over the past several years, many techniques have been developed and applied to overcome these challenges [60]. Nanoparticle tracking analysis (NanoSight) is one of the best method used for exosome size and quantification.

The commonly employed physical characterization methods are microscopy based methods such as transmission electron microscopy, scanning electron microscopy, cryoelectron microscopy and atomic force microscopy [61,62]; dynamic light scattering [63]; nanoparticle tracking analysis [62,64]; tunable resistive pulse sensing [65]; and single EVs analysis [66].

The used molecular methods to analyze the concentration, quantitative and profile of exosomes are quantitative real time PCR [60], digital PCR (chip-based dPCR, droplet digital PCR, ddPCR) [60,67], western blotting, whole genome sequencing (next-generation sequencing) [68], exome-targeted sequencing (next-generation sequencing) [68], microarray profile [69] and ELISA [70].

## Exosome-derived miRNAs as cancer biomarkers

The presence of the tumor at the earliest possible stage (0–1) should be detected by using a sensitive miRNA-based biomarker assay. In addition to tissue biopsy based current studies, investigation of circulating miRNA is a new expanding field in biomarker research because they possess all characteristics (miRNA profiling, diagnosis, prognosis, therapy response and predictive biomarkers), are detectable in liquid biopsy (biological fluids), and do not require both healthy and tumor biopsies from patients. Body fluid such as blood sample enables physicians and researchers to detect the development of cancer at an early stage.

Exosomes have been found to provide a protective and enriched stable source of miRNA in body fluids, preventing their biological molecules from degradation under nonphysiological conditions (multiple freeze-thaw cycles, long-term storage and extreme pH) [71,72]. It has been reported that exosomally derived miRNA remains stable at -20°C for up to 5 years and is resistant to freeze-thaw cycles [17,44,73,74,75]. It makes it a potential biomarker for cancer and other diseases. miRNAs have been implicated in the pathogenesis of many diseases including cancer and have also been shown to be taken up by either distal or nearby recipient cells as cargo in exosomes as a method of cell-to-cell communication to potentially influence the pathogenesis [60,76,77,78,79,80].

miRNAs are known as fundamental regulator of gene expression particularly in cancer, and play an important role in tumorigenesis, metastasis and resistance to various therapies. Over 80.000 articles related to miRNAs in title or keyword have been found by using PubMed [81]. It has been reported that a mammalian cell contains around 100,000 endogenous miRNA molecules per cell [82]. It has also been estimated that a single exosome can carry up to approximately 500 copies of miRNAs [46]. The amount of exosomes in normal human blood has been reported as around 10^9^ exosomes/ml in cancer patients [83,84].

miRNAs are a major class of small, single-stranded, noncoding RNA molecules with a length between 20 and 22 nucleotides (NTs) in their mature form, which play important roles in virtually all biological pathways including cell growth, proliferation, differentiation, immunity response, apoptosis, metabolism and tumorigenesis. The latest release of miRBase (miRBase release v22: http://mirbase.org/) contains 2654 distinct mature miRNAs in humans [81,85], with each potentially having multiple mRNA targets.

**Figure 7.**
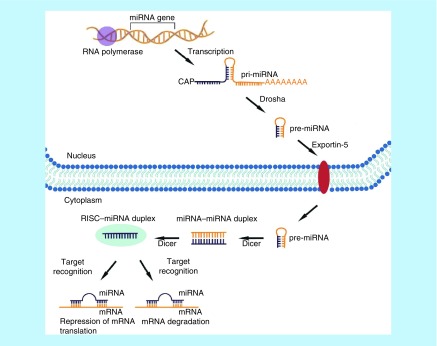
The biogenesis of microRNA. Noncoding miRNAs genes are transcribed in the nucleus into primary miRNAs, which are further processed into premary miRNA and then exported into the cytoplasm where they are finally converted into their matured forms. Mature miRNA then bind to its target mRNA with base pairing, acting as negative regulators of mRNA translation (either mRNA degradation or inhibition of protein expression from mRNA).

miRNA genes in humans and many other organisms are located in varying genomic contexts, which include intergenic (located in between protein coding genes) and intragenic (located in protein coding genes) as short noncoding RNA regions. Human miRNAs are transcribed from the corresponding miRNA genes containing their own promoters (transcribed independently) or intragenically from spliced portions of protein coding genes (transcribed dependently) [86]. This transcription is made with RNA polymerases (Pol) II and III, generating long noncoding primary miRNAs (1–3 kilobases). The primary miRNAs contain one or more miRNAs and are 5′ methyl capped (^7^MGpppG) and 3′ polyadenylated (AAAA.…) tail. These transcripts are further processed in the nucleus by the nuclear RNase III enzyme (DROSHA) and the double-stranded RNA-binding proteins, such as DiGeorge Syndrome Critical Region gene 8 (*DGCR8*), then, leading to premary miRNAs (pre-miRNA, around ≈ 70 NTs in length). After these pre-miRNAs are translocated from nucleus to the cytoplasm through the nuclear pore complex by a nuclear export factor, Exportin-5 (XPO5), they bind to the protein complex of RNase III, DICER and RNA-induced silencing complex, which includes argonaute proteins. DICER cleaves pre-miRNA into a double-stranded RNA of approximately 20–22 NTs in length as miRNA–miRNA dublex. In conjunction with RNA-induced silencing complex, a guide strand (one of the two miRNA strands in the dublex) helps to navigate the mature miRNAs (20–22 NTs) to the target mRNA with base pairing, consequently resulting in downregulation of target gene expression. Intracellular miRNAs are involved in the regulation of gene expression at the post-transcriptional level, acting as negative regulators of mRNA translation by binding to its complementary sequences (usually around 6–8 NTs) in either 5′ untranslated region or 3′ -untranslated region of their target mRNA molecules (Figure 7) [22,87,88,89,90]. The binding of miRNAs to their target mRNAs mainly leads to the mRNA degradation or inhibit expression of target proteins from mRNAs at the post-transcriptional level [78,91].

It is believed that miRNA controls about 60% of all protein-coding genes in human. Among the miRNA–mRNA regulatory relationships, many different miRNAs are often required to act cooperatively to target a single mRNA. On the other hand, a single miRNA can also affect the expression level of multiple mRNAs by targeting a transcription factor [92,93,94]. It is well known that miRNAs play crucial roles in the pathophysiology of many perhaps all human cancers. miRNAs can function either as tumor-suppressors or as oncogenes depending on the target mRNA and play important roles in tumor development, acquisition of drug resistance and metastasis. Many oncogenic miRNAs that have been reported to be aberrantly expressed in different cancer cells are responsible for sustaining a high cell proliferation rate, metastasis and stimulating oncogenic transcription factors [95,96].

Exosomes can be regarded as vehicles for transferring miRNAs to target recipient cells. Exosomes protect miRNAs from degradation, enabling them to be more stable than free miRNAs and to be efficiently integrated by specific recipient cells [97]. Therefore, within the cargo that exosomes carry, miRNAs can provide information about the identity of the cell type from which they are derived, the target and the cellular state, including therapy resistance.

Increasing evidence reveals that tumor cell-derived exosomes have become a central candidate for promoting tumor cell proliferation, invasion, angiogenesis, distant metastasis and remodeling of the tumor microenvironment through transmitting onco-miRNAs [98]. Angiogenesis is essential for malignant tumor growth and metastasis because new blood vessels offer extra oxygen and nutrients and also to remove waste products [99]. It has been demonstrated that cancer cell-released exosomal-miR-21, exo-miR-23; exo-miR-29; exo-miR-103 and exo-miR-210 promote proliferation, angiogenesis and tumor migration [93,94,98,100,101,102,103,104]. Increasing evidence reveals that exo-miR-21 may be a promising biomarker for many types cancer. Pakravan *et al.* reported that exo-miR-10 and exo-miR-100 promote suppression of angiogenesis and downregulation of VEGF in human breast cancer cell model [105,106].

Therefore, tumor-derived exosomes have pivotal roles in cancer progression; especially their miRNA cargoes contribute to manipulating transcriptome pool of target cells. It seems that discovery in the field of exosomal miRNAs biology could uncover the underlying mechanisms promoting the aggressive feature of tumors [98]. Nowadays, 2838 miRNAs have been described in exosomes released from various cell types (FunRich 3.0 released in 2016, www.exocarta.org). However, further studies are required to gain a better understanding of the role of exosomal miRNAs as biomarkers in carcinogenesis and cancer progression.

## Exosomes in clinical applications

Many studies have demonstrated that exosomal miRNAs represent a very promising new therapeutic strategy for human cancer because of their important natural roles in many cellular processes combined with strong stability, tissue specific expression and secretion into all biological fluids [8,17,78,107]. Considering these findings, exo-miRNAs might play an important function during the transformation of normal cells into malignant cells [78]. Studies have identified circulating exo-miRNAs as potential diagnostic and prognostic biomarkers in therapeutic monitoring for cancers [108]. One of the therapeutic strategies of exosome is the inhibition of onco-miRNAs’ expression by delivery of antagonist tumor-suppressive miRNAs for the treatment of cancer. Exosome loaded with therapeutic anti-miRNA oligonucleotides complementary to the sequence of the targeted mature oncogenic miRNAs can be delivered either systemically or through local injection into the tumor. Another therapeutic strategy is the removal of exosomes from the body circulatory system or to prevent the fusion or uptake of exosomes by target cells to inhibit tumorigenesis. Exosome can be isolated from a patient’s fluids and after modification; it can be transferred back to the same patient for targeted cancer therapy [38,109,110].

Recent studies have demonstrated that tumor suppressor miRNA-loaded exosomes can be used against proangiogenetic mRNAs to inhibit tumor angiogenesis. Exosomes are also amenable to be used in genetic therapy, whereby desired therapeutic genetic materials can be delivered to target cells in certain diseases [10,22]. In addition, exosome-delivered some miRNAs can be considered ideal candidates in using specific gene knockdown to inhibit tumor growth.

Exosomes may represent not only the future biomarkers in medicine, but also a very valuable and effective ‘nanovector’ as transport vehicles for delivering targeted anticancer drugs with low immunogenity and toxicity than other drug-delivery vehicles in cancer therapy [7,111].

Since exosomes are small, nontoxic, nonimmunogenic and native to human as their membrane composition is similar to the cells in the body, and it can be used as drug delivery vehicle to the target cells. Drug-loaded exosome-based vehicle can cross the biological barriers, such as the blood–brain barrier, enabling targeted delivery of neuropharmacological agents into the brain. Exosomes isolated from bovine milk were loaded with anticancer agent withaferin A and used against breast and lung cancer in mice models. The study reported a significant higher efficacy of drug loaded into exosome when compared with the free drug [112]. Engineering designs permit the loading of exosomes with miRNAs, siRNAs, genes, small reactive biomolecules, peptides, antioxidants, and ligands, among other strategies to target delivery in cancer [113].

Currently, several clinical trials using exosome-based cancer therapy are ongoing in the worldwide. However, to use exosomes clinically, further research and proper validation are needed to resolve a number of contentious issues such as; purification and characterization in cancer treatment [17,114]. Together, the scope of using exosome is currently limited, likely, utilization of these biomolecules will soon be in place clinically.

## Conclusion & future perspective

The discovery of exosomes as multicomponent signaling complexes mediating cell-to-cell communication between both neighboring (cell-to-cell) and distant cells (travel to distant) is an emerging area as a novel form of communication, as well as a delivery vehicle to carry their cargo. Due to their small size, natural products of the body cells, nontoxic characteristics and crossing the various biological barriers, they are an excellent delivery system for antitumor miRNAs and antitumor drugs in therapeutic tools [22,110,115]. Tumor-derived exosomal miRNA research is highly dynamic and promises novel approaches in cancer prevention, early detection, diagnosis and personalized therapy [17]. In general, there are distinct differences in tumor cell-derived exosomal miRNA expression patterns, compared with their healthy cells. Variations in expression profiles have also been shown to correlate with different tumor characteristics, such as tumor angiogenesis, invasion and metastasis.

It can be possible to inhibit exosome transport proteins such as ESCRT, ALIX and tetraspanins that decreased exosome secretion from cancer cell to neighbor normal cells and this may approach new targets for anticancer therapies to inhibit metastasis. Engineered exosomes could be used to deliver therapeutic agents including anticancer drugs and functional antitumor miRNAs to targeted cancer cells or tissues as personalized cancer therapy. Another possible therapeutic strategy, mature onco-miRNA formation can be inhibited by using inhibitor molecular against to Exportin-5 or DICER proteins as anticancer therapy. As another possible example is also to promote lysosomal degradation of exosomes in tumor cell.

As a result, exosomes are perfectly biocompatible, they reduced toxicity and immunogenity, display great stability in body fluids and can be loaded with specific molecules to targeted cells in cancer treatment. In conclusion, exosomes are small particles with big roles in cancer. Although rapid progress has been made in exo-miRNA detection methods, further efforts to get more sensitive, rapid and cost-effective methods are still needed to find more accurate characterization and functions of exosomal miRNAs from body fluids, thereby providing a strategy for the prevention, early diagnosis and treatment of cancer. This will provide tremendous opportunities for the clinical translation of engineered exosome delivery in targeted cancer therapy in the near future.

**Executive summary**

- Exosomes are small particles with big roles in cancer. They are generated by both normal and tumor cells and are found in all body fluids. The specific exosomes secreted by tumor cells that contain biomarkers can be used to predict the existence of the presence of a tumor in cancer patients.
- The liquid biopsy, a new star of cancer detection, is growing in popularity because of its minimal invasiveness, ease of use, painlessness, lower sample volume, lower cost, higher accuracy and high-throughput.
- Tumor cell-derived exosomal miRNA research is highly dynamic and promises novel approaches in cancer prevention, early detection, diagnosis and personalized therapy.
- Currently, several clinical trials using exosome-based cancer therapy are ongoing. However, to use exosomes clinically, further research and proper validation are needed to resolve a number of contentious issues such as purification and characterization in cancer treatment.
- Further studies are required to gain a better understanding of the role of exosomes in carcinogenesis and cancer progression. This will provide tremendous opportunities for the clinical translation of engineered exosome delivery in targeted cancer therapy in the near future.
